# Initial Implementation of E-Prescribing: Evidence from Eastern Taiwan

**DOI:** 10.3390/healthcare14152285

**Published:** 2026-07-27

**Authors:** Ching-Yu Chang, Chia-Lung Chen, Hui-Ying Chu, Chung-Hung Tsai, Yi-Yuan Chen

**Affiliations:** 1 Medical Affairs Office, Hualien Tzu Chi Hospital, Buddhist Tzu Chi Medical Foundation, Hualien 970, Taiwan; jenny_chang@tzuchi.com.tw (C.-Y.C.); totoro1215@tzuchi.com.tw (H.-Y.C.); 2 Department of Social Policy and Social Work, National Chi Nan University, Nantou 545301, Taiwan; s110103906@mail1.ncnu.edu.tw; 3 Department of Information Technology and Management, Tzu Chi University, Hualien 970, Taiwan; 113221101@gms.tcu.edu.tw

**Keywords:** e-prescribing, access to medicines, digital health service quality, institutional trust, trust in technology, digital trust

## Abstract

**Background/Objectives**: Electronic prescribing (E-prescribing) systems provide several advantages and are a key component of digital health. In Taiwan, the implementation of e-prescribing systems began in 2025. This study aimed to examine health care professionals’ perceptions, satisfaction, trust, and intention to use e-prescribing during its early implementation phase. **Methods**: The research subjects included health care professionals in hospitals and pharmacies in Hualien County, Eastern Taiwan. A total of 107 valid responses were collected and analyzed using structural equation modeling (SEM). **Results**: The access to medicines, digital health service quality, and digital trust significantly positively affected user satisfaction. Both access to medicines and digital trust significantly influenced intention to use. Institutional trust significantly affected digital trust. Additionally, user satisfaction significantly influenced intention to use. Institutional trust indirectly influenced both user satisfaction and intention to use through the mediation of digital trust. Access to medicines, digital health service quality, and digital trust each indirectly influenced intention to use through user satisfaction. **Conclusions**: This study advances the literature on adoption by presenting a novel, technical and social–psychological integration framework that contextualizes e-prescribing within regional health care networks. By validating the key mediating roles of user satisfaction and digital trust, these findings empower health care authorities to mitigate frontline resistance and optimize the implementation of digital health policies.

## 1. Introduction

The World Health Organization (WHO) reported that in recent years, the National Health Service (NHS) in the United Kingdom has developed and implemented digital prescriptions to enhance public health and medical services, producing effective outcomes. Since January 2024, UK citizens have been able to access all prescription information through the NHS mobile app. Users can also review health records, schedule hospital appointments, and designate a preferred pharmacy for prescription dispensing [1]. With the rapid advancement of information and communication technology (ICT), digital or electronic prescribing (e-prescribing) systems have become essential for delivering high-quality and safe health care.

E-prescribing systems are a key component and driving force of digital health. They enhance medication safety and have become increasingly common across health care systems worldwide [2]. These systems strengthen the processes of issuing, transmitting, and processing prescriptions, reducing the risk of errors and optimizing pharmacy management [3]. As global health care systems emphasize patient-centered care, health care administrators must consider how e-prescribing can enhance efficiency and anticipate future trends and challenges in digital health, as these issues significantly impact the quality of medical services. Advances in digital health have created new opportunities in health care, and implementing e-prescribing systems is among the most important steps toward realizing digital health care [4]. Therefore, we explored the factors influencing user satisfaction and intention to use the e-prescribing system among health care professionals. Through empirical analysis, we aimed to offer practical recommendations and policy guidance to optimize and promote e-prescribing systems.

E-prescribing systems can reduce patients’ medication costs and improve access to medicines. Notably, user satisfaction with e-prescribing includes the smoothness of the prescription process, stability of the drug supply, and patients’ ability to obtain appropriate medications with transparent information. Thus, we introduced the concept of access to medicines in this study. Additionally, individuals’ overall evaluation of perceived quality of health care services after using the e-prescribing system will elucidate its effects on user satisfaction and intention to use [5]. Oppong et al. [6] reported that for most health care institutions, a major organizational challenge is providing quality services to an increasing number of patients at the lowest possible cost. Clarifying user satisfaction with the digital health service quality of the e-prescribing system and identifying key contributors to satisfaction and continuous intention to use are therefore valuable. Prior research has suggested that system design flaws, including hardware, workflow, and software issues, contribute to the discontinuation of e-prescribing systems [7]. Conversely, reducing preventable medication errors by issuing clear, legible prescriptions is expected to increase user adoption. Thus, when examining factors affecting e-prescribing implementation, trust in technology must be considered.

Big health care data is an essential component of the medical field, encompassing extensive information about patients, including medical conditions, treatments, and care. However, these data—stored in centralized databases—are vulnerable to security threats, including breaches of patient privacy, data integrity issues, and authentication problems [8]. The loss of such data may disrupt disease diagnosis and treatment processes [9]. Protecting patient privacy in electronic medical records (EMRs) is critical and requires secure data storage and processing methods, as these data contain sensitive personal health information [10]. Balancing professional access to patient information with privacy protection is essential [11]. Consequently, prior research has examined how e-prescribing systems can safeguard information security and patient privacy from a technical standpoint [8]. In line with this, we incorporated institutional trust, an important dimension related to information security and privacy, in this study as one of the key considerations for the successful implementation of e-prescribing systems.

E-prescribing systems allow prescribers to electronically transfer prescription information to the pharmacy’s computer system, mitigating potential prescription and medication errors and reducing the need for pharmacies to contact prescribers for order clarifications. Despite these advantages, many health care providers and pharmacists remain hesitant to fully adopt e-prescribing [12]. In addition, the specific social–psychological barriers and workflow challenges of implementing e-prescribing within the infrastructure of Taiwanese medical institutions remain largely unexamined. To bridge this gap, Eastern Taiwan offers a unique setting: its geographical isolation, dispersed population, and chronic medical personnel shortages provide a rigorous context for digital health adoption. Investigating this environment during its initial deployment phase requires a focus on health care professionals’ trust and satisfaction. In high-stakes clinical environments, institutional and digital trust mitigate perceived technical risks, while user satisfaction is vital to overcoming inertia and ensuring long-term behavioral commitment.

Consequently, this study contributes to the literature by exploring and examining the technical and social–psychological determinants—specifically access to medicines, digital health service quality, and digital trust—that influence user satisfaction and behavioral intention during the inception of e-prescribing in the unique contextual environment of Eastern Taiwan. The findings may provide critical insights and actionable recommendations to refine future policy planning, system design, and managerial strategies, ultimately promoting population health and social well-being.

## 2. Literature Review

### 2.1. E-Prescribing

E-prescribing is the process by which prescribers electronically transmit prescriptions to designated pharmacies via a national system. Within e-prescribing systems, prescriptions are issued, transmitted, stored, distributed, and updated through information networks [13]. The United States Centers for Medicare and Medicaid Services defines e-prescribing as the electronic transmission of prescriptions or prescription-related information, either directly or through an intermediary (such as an e-prescribing network), between prescribers, dispensers, pharmacy benefit managers, or health insurance [14]. Aldughayfiq and Sampalli [2] characterized e-prescribing as the use of electronic devices to deliver and exchange prescription information among stakeholders, including patients, prescribers, pharmacies, and health insurance companies. In summary, e-prescribing is the electronic transmission and exchange of prescription information through devices or networks.

In practice, e-prescribing systems seamlessly integrate with EMRs, allowing physicians to issue and sign electronic prescriptions and transmit them directly to pharmacies. This process ensures the integrity of medical data and enhances prescription accessibility [3]. Additionally, e-prescribing systems may further integrate with telemedicine or teleconsultation systems, leveraging ICT to support the prescribing process and extend existing clinical practices with new functionalities [15]. Building on the digital foundation of EMRs, e-prescribing systems represent a key technology that can advance medication management transformation. E-prescribing has become an integral part of modern health care; it improves medication management and facilitates the implementation of telemedicine [16]. Its advantages include enhancing access to medications through easier prescription renewals without in-person visits, reducing costs associated with prescription renewals, and allowing prescribers to access complete medication histories via a centralized digital prescription database [17].

Despite significant investments and widespread availability, the clinical utilization of e-prescribing systems for initial prescriptions and refills remains below expectations. Additionally, implementing e-prescribing faces complex, multidimensional challenges [18,19]. Gagnon et al. [18] identified key facilitators of e-prescribing implementation perceived by stakeholders, including technical design, interoperability, tailored content, user attitude, productivity, and infrastructure resources. Recent empirical evidence delineates four critical typologies of implementation barriers: technical system limitations, workflow misalignments, clinician behavioral factors, and institutional or regulatory constraints [19]. Ebad [20] demonstrated that the determinants of health information technology (HIT) implementation failure are divergent, encompassing technical and managerial dimensions. Notably, suboptimal training administration—specifically regarding timing and duration—alongside systemic integration deficits with existing enterprise systems emerged as the most critical catalysts for failure, followed by deficiencies in functional software design and human resource management. Therefore, when system designers neglect end-user involvement, workflow adaptation, and clinical needs during development, the resulting platform suffers from architectural inflexibility, poor usability, and resistance from frontline users, ultimately compromising patient safety and well-being [21].

While e-prescribing systems are established in several advanced countries, their formats and levels of automation vary across nations [3]. In the US, e-prescribing occurs over the internet and integrates with both EMR and pharmacy systems. Data security and confidentiality standards adhere to the Health Insurance Portability and Accountability Act, enacted in 1996, which sets regulations for information security and privacy. The Health Information Technology for Economic and Clinical Health Act, enacted in 2009, further established encryption standards and specified certain technical requirements [22]. E-prescribing has also been incorporated into the overall health care system to enable subsequent collection and analysis of pharmacotherapy data and patient-related information. In the UK, e-prescribing is integrated into NHS, allowing patients to choose a pharmacy for dispensing [3]. UK citizens can access all prescription information through the NHS mobile app. Additionally, users can review their health records, schedule hospital appointments, and designate a preferred pharmacy for prescription dispensing [1]. In Sweden, e-prescribing has been implemented for many years and is regarded as highly successful. The Swedish eHealth Agency, responsible for promoting EMRs, electronic prescriptions, data standardization, and AI governance, exemplifies Sweden’s comprehensive digital health governance framework [23].

Digital transformation in health care has become an inevitable trend in advanced nations. In Taiwan, to improve drug safety and reduce medication duplication, the Taiwan National Health Insurance Administration (NHIA) launched the “PharmaCloud” system in 2013, an electronic platform that allows authorized prescribers and pharmacists to access a patient’s comprehensive medication profiles across different institutions [24]. Huang et al. [25] demonstrated that using the modified WHO protocol with data from the PharmaCloud significantly reduced the time required to obtain the best possible medication history (BPMH) while substantially enhancing the system’s error detection capabilities during hospital admission and discharge. Meanwhile, patient-centric mobile health (mHealth) applications have evolved into integrated platforms for electronic prescribing and medication inquiry. Chen et al. [26] highlighted that user experience and information impact user ratings of drug reference apps. A pilot study demonstrated the value of a novel mHealth approach for individualized medication and health management in Taiwan [27].

Serving as a landmark upgrade to the foundational 2013 ‘PharmaCloud’ policy, the NHIA officially launched its comprehensive e-prescribing system initiative in 2025. In alignment with efforts by the Ministry of Health and Welfare to standardize EMR data, FHIR (Fast Healthcare Interoperability Resources) has been introduced as the standard data format along with QR codes, enabling prescription transmission through the “My Health Bank” platform and the “Real Name Verification for Medical Institutions” mobile app. The objectives are to reduce paper use and health care costs, enhance medication safety, improve data utilization efficiency, and lower environmental carbon emissions [28]. In 2024, the NHIA commissioned Hualien Tzu Chi Hospital to initiate an e-prescribing system project among health care institutions in Eastern Taiwan, marking the first implementation of e-prescribing in Taiwan. This study surveyed health care professionals involved in this e-prescribing system project and validated the proposed research framework, providing a reference for subsequent policy formulation and program implementation.

While the prior e-prescribing literature overlooks localized medication accessibility and multidimensional digital trust in resource-constrained mandates, this study addresses this gap through a structural equation modeling (SEM) analysis of Taiwan’s 2025 national e-prescribing initiative in the regional city of Hualien. By anchoring “access to medicines”, “institutional trust”, and “trust in technology” within a technical and socio-psychological framework, this research transcends conventional adoption models by validating user satisfaction as a crucial mediating mechanism. This model elucidates how access to medicines, digital health service quality, and digital trust concurrently drive clinician behavioral intention, yielding transferable insights for health care systems globally.

### 2.2. Access to Medicines

WHO defines access to medicines as ensuring that medicines are consistently available within a functioning health system, in appropriate amounts, proper dosage forms, assured quality, and adequate information, at prices affordable to individuals and communities [29,30]. Afzali et al. [31] noted that access to medicines is essential for achieving health and affects societal well-being. Access to medicines consists of three principal objectives: physical, economic (or affordability), and informational accessibility. Physical accessibility refers to the availability and geographic accessibility of medicines for reasonable use by those in need. Affordability refers to an individual’s ability to pay for medicines without incurring financial hardship. Informational accessibility encompasses the right to request, obtain, and convey information. Improving access to medicines is vital for enhancing population health and well-being and constitutes a primary goal of all health care systems [31]. Its scope extends beyond the availability of physical medicines to include financial affordability and information adequacy. In this study, access to medicines is defined as professionals’ perceived improvement in access to medicines.

E-prescribing systems have the potential to enhance patients’ access to prescription medicines and increase convenience [32]. These systems also improve health care professionals’ access to medicines, promoting user satisfaction and intention to use. At the point of care, e-prescribing enables prescribers to transmit accurate, error-free, and understandable prescriptions electronically to pharmacies. Nurses use e-prescribing systems for medication management, while pharmacies review prescription content and manage medicine inventories [15]. Moreover, they enhance the efficiency of health care services, reduce costs, improve patient safety, save time for physicians, pharmacists, and patients, and enable monitoring of prescription issuance to prevent misuse and overprescribing [33,34,35,36].

Another advantage of e-prescribing systems is that they provide physicians with comprehensive information on patients’ medication histories through centralized electronic prescription databases, preventing excessive medication use and related health risks [17]. By effectively facilitating access to medicines, e-prescribing mitigates communication barriers within medical processes and improves overall health care service efficiency. These positive experiences reduce health care professionals’ workload and increase workflow efficiency, contributing to higher satisfaction [37]. Furthermore, when medical records and medication histories are integrated in e-prescribing systems, health care workers can quickly understand patients’ overall medication status and avoid drug–drug interactions and misuse [8,38]. This enhances the perceived usefulness of e-prescribing systems, thereby strengthening the intention to continue using the system [39]. Similarly, Alipour et al. [13] indicated that by enhancing access to medicines, e-prescribing effectively increases patient satisfaction, strengthens physician–patient relationships, and enhances medication adherence and active engagement in health care. Consequently, e-prescribing systems facilitate clinical workflows, reduce paper consumption, and improve the efficiency and effectiveness of clinical decision-making. These improvements reflect enhanced patient safety, workflow efficiency, and overall operations. At the same time, issues from paper-based prescriptions, such as medication errors, inefficiency, and increased health care costs, are eliminated [38]. These positive effects further influence user satisfaction and intention to use among health care professionals. Therefore, we hypothesized as follows:

**H1a.**  *Access to medicines exerts a significant positive effect on user satisfaction.*

**H1b.**  *Access to medicines exerts a significant positive effect on intention to use.*

### 2.3. Digital Health Service Quality

According to Kruk et al. [40], high-quality health care systems could prevent approximately 2.5 million cardiovascular deaths, one million neonatal deaths, 900,000 tuberculosis deaths, and half of all maternal deaths annually. Conversely, poor-quality health care may lead to adverse health outcomes. Therefore, evaluating the quality of health care is crucial.

As patient-centered health care gains attention, evaluating health service quality from the patient’s perspective has become increasingly important. The Institute of Medicine defines health service quality as the degree to which health services for individuals and populations increase the likelihood of desired health outcomes and align with current professional knowledge [41]. This definition emphasizes that expected health outcomes reflect not only patient satisfaction and well-being but also subsequent health status or quality of life. The European Commission [42] and WHO [43] have both stressed that high-quality health services should be effective, safe, and people-oriented.

In recent years, the emergence of Industry 4.0, or the Fourth Industrial Revolution, has led to the integration of new ICTs in organizations. This integration facilitates and supports more efficient and flexible processes, services, and products, ultimately driving organizational digital transformation [44]. Similar to the manufacturing sector, the health care industry has undergone digital transformation and evolution over the past few decades, known as Health Care 4.0. Health care 4.0 is a data-driven system that incorporates technologies such as digital health [45]. Digital health encompasses the application of digital technologies in the health care context, including eHealth, mHealth, and telehealth [46]. It refers to the use of ICTs to enhance health, health care services, and the well-being of individuals and populations [47]. The literature suggests that digital health service quality can be summarized as individuals’ overall evaluation of perceived health care quality after using digital health services [6,45,48].

In their study of e-commerce platforms, Ashiq and Hussain [48] found that e-service quality influences users’ e-satisfaction, which, in turn, affects their e-loyalty. A prior study indicated that, in developing countries, digital health service quality is a key determinant of public acceptance [49]. Therefore, to implement e-prescribing systems, governments and service providers must prioritize ensuring the quality of digital health services [50,51]. These findings align with those of Oppong et al. [6], who suggested that digital health service quality significantly positively impacts user satisfaction and intention to use. Within health informatics, there is a strong consensus on the critical relationship between the quality of digital health services and users’ behavioral intention to use them [5]. Empirical evidence demonstrates that robust service quality safeguards data integrity and ensures operational continuity during hospital-to-pharmacy transitions [25]. By delivering reliable technical performance and seamless interface experiences, high service quality fosters institutional digital trust among clinicians, reassuring them of the tool’s pragmatic utility. Thus, ensuring premium service quality is not merely a technical objective but a practical prerequisite for transforming initial system deployment into sustained, long-term utilization. In the study, we hypothesized as follows:

**H2a.**  *Digital health service quality exerts a significant positive effect on user satisfaction.*

**H2b.**  *Digital health service quality exerts a significant positive effect on intention to use.*

### 2.4. Institutional Trust

Institutional trust establishes trust in online environments. It is defined as trust arising from assurances of safety, secure networks, or structural safeguards inherent to a given context, unrelated to any specific individual [39]. Institutional trust reflects individuals’ perceptions or beliefs that the transaction environment provides appropriate security and protection [52].

Information security and privacy are critical elements of institutional trust. Hoffman et al. [53] argued that information security and privacy are key drivers of online trust, reporting that the perceived control over vendors’ actions directly shapes consumers’ perceptions of online information security and privacy. Gefen et al. [39] suggested that institutional trust emerges from individuals’ sense of security within a given context, driven by guarantees, secure networks, or other institutional structures. In online contexts, trust cues include certification seals and explicit privacy policy statements. A recent study in digital health care indicated that institutional safeguards reflect the impact of the external environment of digital health systems (e.g., telemedicine) on health care service delivery. Indeed, institutional safeguards significantly affect online trust [54].

Multiple studies have shown that institutional trust significantly influences trust beliefs and intentions to trust in online environments [52,55]. Enhancing the security features of e-prescribing systems can strengthen user trust [8]. Prior research noted that information security and privacy are major barriers to the widespread adoption of e-prescribing systems [12]: patient information may be leaked, and protected health information could be stolen without proper firewalls or intrusion detection systems. Thus, ensuring the security of patients’ personal medical information is critical for electronic information systems [10,11]. Consequently, institutional trust is an important factor for digital trust. Therefore, we hypothesized the following:

**H3.**  *Institutional trust exerts a significant positive effect on digital trust.*

### 2.5. Trust in Technology

Trust in technology refers to individuals’ beliefs in the specific attributes of a technology. Beliefs regarding trust in a technology involve functionality, helpfulness, and reliability [56]. Kuen et al. [57], based on Stewart’s [58] trust transfer theory, explained that trust can be transferred from a known entity to a new, unfamiliar entity. In forming beliefs about an unfamiliar entity, individuals rely on their trust in a known entity as a cue for judgment. Thus, interpersonal trust among health care professionals can extend to unfamiliar digital technologies they use (e.g., telemedicine or e-prescribing systems), constituting their trust in technology.

Previous research indicates that prescribing errors are a preventable source of harm in health care. Such risks can be mitigated through e-prescribing systems, which enable physicians to use automated, digitally assisted systems to identify potentially inappropriate prescriptions (e.g., drug allergies) [59]. Therefore, leveraging ICTs within e-prescribing systems to reduce errors is critical for securing user trust. Trust in technology is essential for e-prescribing systems [8]. Consequently, numerous studies have examined ways to address technical issues in these systems to strengthen digital trust [8,49,60]. In our study, we hypothesized as follows:

**H4.**  *Trust in technology exerts a significant positive effect on digital trust.*

### 2.6. Digital Trust

Digital trust refers to stakeholders’ confidence in actors, technologies, and processes that establish reliable and secure enterprise networks [61]. Kozhevnikov and Korolev [62] conceptualized digital trust as the relationship between an individual and an independent intelligence agent within a digital environment [63]. In the era of AI, the use of intelligent agents in health care has become increasingly common, with applications such as virtual assistants, chatbots, telemedicine, and e-prescribing. However, the privacy and security of patients’ personal information remain significant challenges [64]. Igwe-Nmaju and Anadozie [65] noted that advanced digital technologies, now embedded in daily life, have transformed the trust relationship between organizations and stakeholders. This new trust dynamic has become a key determinant of organizational legitimacy. They suggested that digital trust reflects stakeholders’ confidence in an organization’s reliability, ethical conduct, and security in delivering services through digital systems [65]. The core technologies and mechanisms of e-prescribing systems include personal identity authentication and digital signatures [66]. These technologies are specific applications of digital public infrastructure, which refers to foundational, reusable digital platforms such as digital ID, digital payments, and data sharing. This infrastructure is regarded as a key element in establishing digital trust [67].

Users’ trust in e-prescribing systems is crucial for their satisfaction and behaviors [59]. In previous attempts to digitize health information, implementation failures often stemmed from insufficient confidence in the system [68]. Thus, enhancing system security and strengthening trust are critical issues for e-prescribing systems [8]. In the context of health information digitalization, trust consistently emerges as a focal point, determining whether the system can yield positive outcomes for users [69]. Additionally, evaluation results can enhance the intention to use the system [70]. In this study, we hypothesized the following:

**H5a.**  *Digital trust exerts a significant positive effect on user satisfaction.*

**H5b.**  *Digital trust exerts a significant positive effect on intention to use.*

### 2.7. User Satisfaction and Intention to Use

User satisfaction refers to the favorable outcome perceived by users or service seekers from interactions with service providers [71]. Previous research indicates that in adopting health insurance services, user satisfaction affects subsequent intentions to use [70]. In digital information or telemedicine systems, user satisfaction significantly positively influences continued intention to use [72,73]. Additionally, Ashiq and Hussain [48] found that e-service quality influenced users’ e-satisfaction, which, in turn, affected their e-loyalty. Therefore, we hypothesized the following:

**H6.**  *User satisfaction exerts a significant positive effect on intention to use.*

## 3. Materials and Methods

### 3.1. E-Prescribing Systems

The E-prescribing system evaluated in this study was developed by Hualien Tzu Chi Hospital (Hualien City, Taiwan). It transforms the traditional paper-based prescription workflow into a standardized, exchangeable, and traceable electronic process. The system comprises four core functional modules:

Prescription Issuing Module: Retrieves prescription data from the internal HIS (Hospital Information System), converts it into FHIR format compliant with electronic prescription exchange fields, and generates the prescription QR Code and digital signature.

Dispensing Module: Enables pharmacies to query prescriptions, download FHIR-formatted prescription data, upload dispensing annotations, query the number of times a prescription has been dispensed, and upload dispensing records.

Authentication and Digital Signature Module: Obtains verification information and digital signatures via a Secure Module Card (SAM card) or a Cloud Secure Module, ensuring proper institutional identity verification for upload and query operations.

Response and Error Handling Module: Decodes transaction results, tracks logs, and handles errors based on the RtnCode (Return Code), RecvSeqNo (Receiving Sequence Number) and exception codes returned by the Web API (Application Programming Interface).

**Figure 1:**
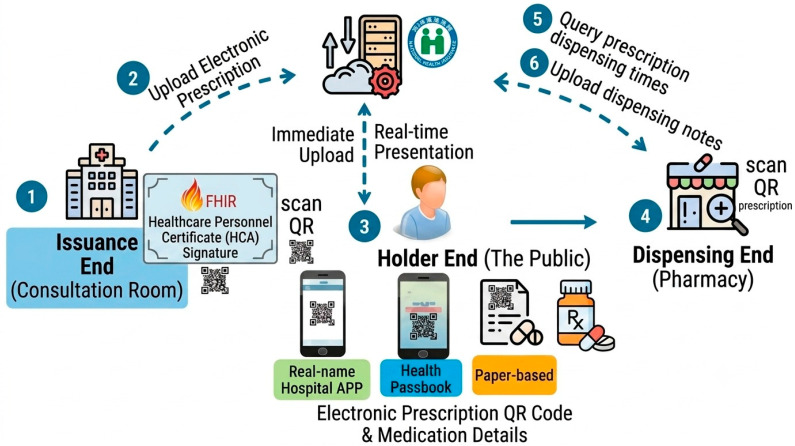
The e-prescribing and dispensing processes.

The e-prescribing and dispensing processes comprise four phases: (1) Issuance: Prescription data are converted into the FHIR standard and uploaded to the NHIA server; (2) QR Code Generation: A QR code is generated, accessible via the NHIA My Health Bank app, institutional mobile apps, or a paper format; (3) Retrieval: Pharmacies scan the QR code to parse prescription details—supporting offline reading—and query the server to verify dispensing eligibility; and (4) Execution: The NHIA Web API is invoked to check prior dispensing counts for validation, followed by medication dispensing and the upload of dispensing records, as shown in Figure 1.

Following the pilot implementation of the e-prescribing initiative in this study, technical verification has confirmed its feasibility. The NHIA is currently promoting this innovative system, aiming to gradually expand its implementation across counties, regions, and institutions, including hospitals at all levels, clinics, public health centers, and pharmacies, to ultimately achieve the vision of completely paperless prescriptions.

### 3.2. Research Framework

**Figure 2:**
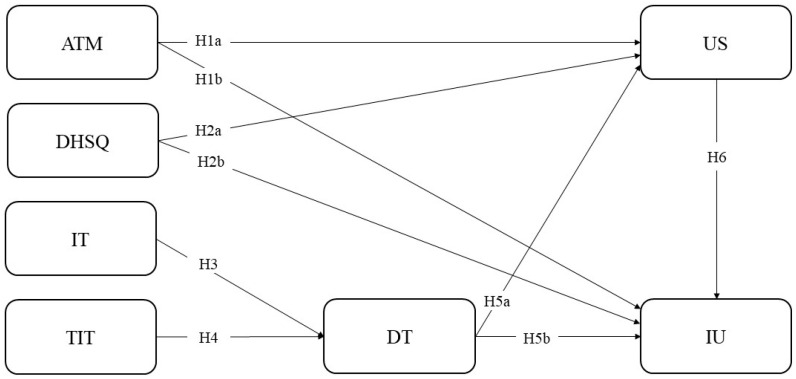
Proposed research framework. Note: ATM: Access to Medicines; DHSQ: Digital Health Service Quality; DT: Digital Trust; US: User Satisfaction; IU: Intention to Use; IT: Institutional Trust; TIT: Trust in Technology.

The research framework of this study is illustrated in Figure 2. We aimed to examine factors influencing health professionals’ intention to use e-prescribing systems. The framework involves observing correlations among two or more variables, including their strengths and directions. This study explored the effects of access to medicines, digital health service quality, and digital trust on user satisfaction and intention to use. Moreover, we examined whether institutional trust and trust in technology influenced user satisfaction and intention to use through digital trust as a mediating variable. Lastly, we investigated the correlation between user satisfaction and intention to use.

### 3.3. Questionnaire Design

We employed measurement scales from the prior literature aligned with the research context. Each questionnaire item was reviewed and revised by experts and scholars specializing in health information technology and health care management to ensure precision, readability, and accuracy. The questionnaire consisted of two parts. The first part measured demographic variables (e.g., sex, age, marital status, educational level, living arrangements, and annual income). The second part included measurement scales for access to medicines, digital health service quality, digital trust, institutional trust, trust in technology, user satisfaction, and intention to use. All items were scored on a five-point Likert scale, where 1 = “strongly disagree” and 5 = “strongly agree.” A total of 24 items were included in this part.

Access to medicines was measured using three items adapted from scales developed by Oppong et al. [6], Guijarro et al. [74], and Hareem et al. [16]. Digital health service quality was measured using three items adapted from scales by Guijarro et al. [74] and Liu et al. [54]. User satisfaction was measured using three items from scales developed by Guijarro et al. [74] and Sema et al. [38]. Intention to use was measured using three items adapted from scales by Kuen et al. [57] and Liu et al. [54]. Institutional trust was assessed using two items adapted from scales by Gefen et al. [39] and Liu et al. [54]. Trust in technology was measured using three items adapted from scales developed by Velsen et al. [75], Teferi et al. [76], Kuen et al. [57], and Sema et al. [38]. Digital trust was measured using four items adapted from scales by Velsen et al. [75] and Liu et al. [54].

This study was conducted at Hualien Tzu Chi Hospital in Taiwan. The participants in this study were recruited from health care institutions in Hualien County that had volunteered to participate in the e-prescribing pilot program, encompassing six medical institutions and eleven community pharmacies. The recruitment procedure involved engaging health care professionals in a hands-on simulation of the e-prescribing workflow, which spanned the clinical consultation, payment, and medication dispensing phases, alongside accessing relevant prescription information via a mobile application. A total of 107 professionals completed this recruitment process. Upon signing the informed consent form, all 107 individuals enrolled as formal participants in this study, yielding a 100% response rate for the subsequent questionnaire (Table 1). This study was part of the NHIA’s 2024 pilot program for e-prescribing systems. Data collection spanned from 17 January to 10 March 2025. The study received approval from the Research Ethics Committee of Hualien Tzu Chi Hospital on 17 January 2025 (Approval No.: IRB114-005-B). To mitigate potential history effects and temporal biases, institutional records were audited during the data collection period. It was confirmed that the core infrastructure architecture and national regulatory frameworks remained stable, ensuring that no significant system updates or exogenous confounding variables had distorted user behavioral patterns.

The prior literature emphasizes that the case-to-parameter ratio method for estimating the required sample size serves as a general guideline, as minimum sample requirements depend on factor loading magnitudes and model complexity [77]. The target population comprised health care institutions participating in the initial pilot phase of the Taiwanese government’s e-prescribing initiative, which was localized to Hualien County, Eastern Taiwan, and operated voluntarily. The absence of external financial or institutional incentives restricted the attainable sample size. Given these specific environmental contexts and the novelty of the target system, it was unfeasible to gather an extensive sample volume as recommended by Kline [78].

To justify the adequacy of the empirical sample size (N = 107) for conducting covariance-based SEM under these constraints, a post hoc statistical power analysis was performed using the framework proposed by MacCallum et al. [79] and implemented via modern psychometric adaptations [80]. With substantial degrees of freedom (df = 157) at α = 0.05, the analysis yielded a statistical power of 0.875 for testing the close-fit hypothesis (H_0_: RMSEA ≦ 0.05 versus H_1_: RMSEA = 0.08). This exceeds the standard threshold of 0.80 [81], indicating that the obtained sample size provides sufficient empirical stability and robustly safeguards the resulting path coefficients against model overfitting vulnerabilities.

### 3.4. Statistical Analysis

Statistical analysis was conducted in three parts. The first part involved descriptive statistics, reliability analysis, and exploratory factor analysis of the demographic variables and measurement scales using SPSS 28.0. The second part examined the relations among the seven variables through confirmatory factor analysis (CFA) and structural equation modeling (SEM) in AMOS 28.0. The third part involved mediation effect analysis using SPSS Process Macro 4.2.

## 4. Data Analysis and Results

### 4.1. Descriptive Statistical Analysis

All questionnaires were fully completed, yielding a 100% valid response rate. Females constituted the majority of the cohort (71.0%), while males accounted for 29.0%. The most prevalent age group among respondents was 40–49 years (35.5%), followed by those aged 30–39 years (25.2%). Most participants were married (65.4%), with unmarried individuals representing 29.0%. Regarding educational level, most respondents held a university (college) degree (82.2%), while 17.8% held a postgraduate degree. Participants living with a spouse and children represented the majority (32.6%), followed by those living with parents (23.5%). Most participants had an annual salary of NTD 200,001–500,000 (37.4%), followed by those earning NTD 500,001–700,000 (22.4%). Professionals with more than 15 years of work experience made up the majority (80.4%), with those having 10–15 years of experience comprising 15.9%. In terms of ease of computer use, the majority found it “easy” (39.3%), followed by those considering it “very easy” (31.8%). The most common prescription type was one-time prescriptions, self-dispensing (42.1%), followed by refill prescriptions (2–3 months), first-time self-dispensing (14%). General practice accounted for the largest share of clinical departments (38.3%), followed by “others” (26.2%).

### 4.2. Common Method Variance (CMV) Analysis

To rigorously assess the potential threat of Common Method Variance (CMV), this study employed the Unmeasured Latent Method Construct (ULMC) approach as recommended by Podsakoff et al. [82]. A baseline measurement model containing only the theoretical constructs (Model A) was compared against an alternative model (Model B) that included a single unmeasured latent method factor. Following the statistical specifications outlined by Williams and Anderson [83], the method factor was constrained to be orthogonal to all theoretical constructs, its variance was fixed at 1, and its paths to all manifest items were constrained to be equal to ensure model identification.

The baseline model (Model A) demonstrated an acceptable fit to the empirical data: RMR = 0.03, GFI = 0.75, NFI = 0.90, and CFI = 0.94. Upon introducing the unmeasured latent method factor, the overall model fit of Model B remained virtually identical to Model A, with GFI, NFI, and CFI unchanged (GFI = 0.75, NFI = 0.90, CFI = 0.94). Meanwhile, the Root Mean Square Residual (RMR) increased negligibly from 0.03 to 0.04.

The integration of the method variance control factor did not improve descriptive fit indices. It slightly increased the unexplained residual variance, indicating that forcing items to share a single method variance structure does not enhance the model’s explanatory power. Consequently, these statistical findings provide empirical evidence that common method variance is not a significant threat in the current dataset, validating the robustness of the subsequent hypothesis testing.

### 4.3. Reliability and Validity Analysis

#### 4.3.1. Convergent Validity Analysis

Based on the recommendations of Hair et al. [84], we assessed convergent validity by calculating composite reliability (CR) for all latent variables and average variance extracted (AVE) for each latent variable via CFA. The factor loadings for all items ranged from 0.651 to 0.988, and the squared multiple correlations for the measurement variables ranged from 0.424 to 0.976. All items met the criterion proposed by Jöreskog and Sörbom [85], namely that the square multiple correlation values should exceed 0.20. Additionally, as shown in Table 2, the CR values for each construct consistently exceeded 0.7, and all AVEs exceeded 0.5, indicating good internal consistency between the latent and observed variables. Overall, all constructs demonstrated good convergent validity.

The item “Patients can obtain their medications at the pharmacy through e-prescribing” exhibited a factor loading of 0.651 and a Squared Multiple Correlation (SMC) of 0.424. This modest statistical performance can be attributed to the study’s timing during the pilot phase of the national initiative, which was characterized by ongoing cross-institutional system integration. Consequently, medical institutions implemented a dual-track configuration where paper and electronic prescriptions coexisted, and information-exchange mechanisms between hospitals and community pharmacies had not yet fully matured. Furthermore, since not all electronic prescriptions were universally applicable for dispensing at community pharmacies at this initial stage, the item’s factor loading and SMC value were inherently constrained.

#### 4.3.2. Discriminant Validity and Reliability Analysis

We evaluated discriminant validity based on the criteria proposed by Fornell and Larcker [86]: (1) the correlation coefficient between any two constructs must be less than 1; (2) the squared correlation coefficient between any two constructs must be smaller than the Cronbach’s α of each construct; and (3) the correlation coefficient between any two constructs must be smaller than the square root of the AVE for each construct. Meeting these criteria indicates sufficient discriminant validity between constructs.

Based on the CFA results, all constructs initially met the first two assessment criteria. However, the square root of the Average Variance Extracted (AVE) for Trust in Technology (0.92) was lower than its correlation with Digital Trust (0.95), thereby violating the Fornell and Larcker criterion. To resolve this overlap and mitigate potential multicollinearity, Trust in Technology and Digital Trust were merged into a single construct labeled “Digital Trust” (Table 3). Following this consolidation, the revised construct successfully met the discriminant validity requirements, with its inter-construct correlations falling well below the square root of its AVE (0.94). Furthermore, the merged construct “Digital Trust” yielded a Cronbach’s α of 0.981, a CR of 0.981, and an AVE of 0.883. These values satisfied the standard criteria of a CR greater than 0.7 and an AVE greater than 0.5.

The discriminant validity issue between trust in technology and digital trust under the Fornell and Larcker criterion may be attributed to several factors. During the inception of e-prescribing, health care professionals served as the primary user group. Given the direct relevance of e-prescribing to their clinical workflows, these early adopters likely evaluated digital trust through the lens of technology features, leading to a conceptual overlap and a high correlation between the two constructs. Furthermore, because this study was conducted during the pilot phase of the initiative, the population of health care professionals with hands-on e-prescribing experience was inherently constrained, making the empirical distinction between trust in technology and digital trust less pronounced during this initial stage.

In terms of reliability, we assessed the internal consistency of each construct using Cronbach’s α. Ranging from 0.893 to 0.981, all Cronbach’s α exceeded the 0.7 threshold recommended by Nunnally [87], indicating that our measurement scales exhibited strong internal consistency reliability.

### 4.4. Path Analysis of the Research Model

**Figure 3:**
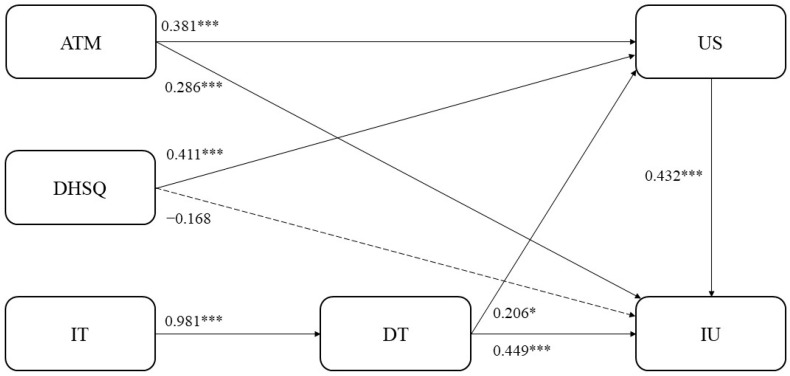
Structural model analysis results. Note: ATM: Access to Medicines; DHSQ: Digital Health Service Quality; DT: Digital Trust; US: User Satisfaction; IU: Intention to Use; IT: Institutional Trust; * *p* < 0.05; *** *p* < 0.001.

The goodness-of-fit indices for the research model were χ^2^/df = 1.615, GFI = 0.822, RMSEA = 0.076, NFI = 0.938, CFI = 0.975, RFI = 0.920, IFI = 0.976, TLI = 0.968, PNFI = 0.724, and PGFI = 0.562. The majority of the fit indices satisfied the recommended benchmarks [81,82], indicating that the structural model exhibited a reasonable and acceptable fit to the empirical data. Subsequent verification of the structural model revealed that, except for H2b, all proposed hypotheses were supported, as shown in Figure 3 and Table 4. Specifically, access to medicines, digital health service quality, and digital trust exerted significant positive effects on user satisfaction. Both access to medicines and digital trust exerted significant positive effects on intention to use. Institutional trust and trust in technology exerted significant positive effects on digital trust. Lastly, user satisfaction had a significant positive impact on intention to use.

### 4.5. Mediation Analysis of the Research Model

Based on Model 4 of the SPSS Process Macro 4.2, we employed bootstrapping to test indirect effects. We calculated 10,000 bootstrap samples to estimate the mediating effects along each predictive pathway. If the 95% confidence interval (CI) for an indirect effect contains 0, the effect is non-significant, indicating the absence of mediation. Conversely, if the 95% CI for an indirect effect does not include 0, the effect is significant, indicating mediation. If the 95% CI for the direct effect includes 0, the direct effect is non-significant, indicating full mediation. Alternatively, if the 95% CIs of both the indirect and direct effects do not include 0, and the 95% CI of the total effect does not include 0, then partial mediation is indicated. The results are shown in Table 5.

Regarding digital trust, (1) for the pathway of “institutional trust → digital trust → user satisfaction,” the indirect effect was significant, while the direct effect was non-significant, indicating full mediation; (2) for the pathway of “institutional trust → digital trust → intention to use,” the indirect effect was significant, and the direct effect was non-significant, indicating full mediation. Institutional trust indirectly influences both user satisfaction and usage intention through the mediation of digital trust, demonstrating statistically significant indirect associations.

Regarding user satisfaction, (1) for the pathway of “access to medicines → user satisfaction → intention to use,” both the indirect and direct effects were significant, indicating partial mediation; (2) for the pathway of “digital health service quality → user satisfaction → intention to use,” the indirect effect was significant, while the direct effect was non-significant, indicating full mediation; and (3) for the pathway of “digital trust → user satisfaction → intention to use,” both the indirect and direct effects were significant, indicating partial mediation. Access to medicines, digital health service quality, and digital trust each indirectly influence intention to use through the mediation of user satisfaction, demonstrating statistically significant indirect associations.

The mediation analysis indicates that digital service quality indirectly influences usage intention through user satisfaction. The path analysis reveals a full mediation effect, suggesting that enhancing digital service quality alone is insufficient to significantly drive health care professionals’ intention to adopt electronic prescriptions; rather, this objective must be achieved by cultivating user satisfaction. Consequently, IT service personnel must treat health care professionals as key stakeholders in customer relationship management during the design and implementation of electronic prescription systems, actively listening to their needs and addressing their problems and barriers. Additionally, government agencies should provide rewards and incentives to encourage participating medical institutions and personnel to demonstrate positive attitudes, thereby fostering their long-term commitment to digital transformation initiatives.

## 5. Discussion

### 5.1. Managerial Implications

This study explored the initial implementation experience of e-prescribing systems and factors affecting their effectiveness. The factors analyzed included access to medicines, digital health service quality, institutional trust, trust in technology, digital trust, user satisfaction, and intention to use. The empirical findings confirmed that access to medicines positively influenced both user satisfaction and intention to use.

Prior research indicates that the primary motivations for adopting e-prescribing are the convenience of obtaining medications [7] and the reduction of errors occurring between the point of care and the pharmacy [15]. A smooth prescription process and stable medicine supply enhance patients’ convenience in medication collection, thereby improving user satisfaction and intention to use. Furthermore, when prescriptions are transferred directly from medical institutions to pharmacies via e-prescribing systems, overall health care service efficiency increases, streamlining the process. This leads to higher satisfaction levels among health care professionals with e-prescribing systems, fostering greater willingness to continue using them. Thus, access to medicines serves as a key driver for the acceptance of e-prescribing systems.

From a practical implementation perspective, we recommend that the government provide various incentives and reward programs to encourage medical institutions and pharmacies to adopt e-prescribing systems, thereby increasing overall usage rates. Additionally, establishing support measures for convenient medication collection, such as sending SMS reminders and offering services like pharmacy callouts and home delivery, can enhance access to medicines, ultimately improving public satisfaction and the intention to continue using the system.

Our study showed that digital health service quality significantly enhances user satisfaction but not intention to use. This may be due to the focus on aspects such as responsiveness and reliability, including whether physicians and pharmacists can clearly address patients’ questions through e-prescribing systems. Although e-prescribing systems improve satisfaction during use, lingering doubts about their responsiveness to clinical issues or ability to integrate medical processes may weaken their impact on intention to use. From the perspective of frontline users, digital health service quality boosts user satisfaction, particularly in terms of system responsiveness and interface clarity, by facilitating daily operations.

However, this construct emphasizes performance based on current system functions. Factors such as the system’s ability to manage cross-department or cross-institutional prescription processes or support digital health care scenarios can also affect users’ intentions to use the system. Consequently, while digital health service quality enhances the operational experience, inadequate institutional or process-level support may hinder its influence on intention to use. From a practical implementation perspective, the rapid emergence of new technologies often elicits psychological resistance among health care professionals, as implementation requires adapting workflows, changing existing routines, and addressing potential risks related to medical disputes. Therefore, training programs and policy communication during early implementation stages are essential actions for both the government and medical institutions to promote. Since health care professionals must provide thorough explanations and guidance to first-time users of e-prescribing systems, the government should organize public policy briefings and informational sessions at medical institutions or township health centers. Additionally, the government should assign dedicated staff to offer consultations at local health departments and establish chatbots or FAQ sections on official websites to address public inquiries, thereby facilitating the comprehensive adoption of e-prescribing systems.

Institutional trust is a key contributor to the adoption of e-prescribing systems. Ensuring the security and privacy of these systems is essential [12], as they handle sensitive patient information and identity verification [8]. Institutional trust positively influences user satisfaction and intention to use through digital trust. If e-prescribing systems fail to securely protect patient information, such as diagnoses, prescribed medications, and personal identity, patients may lose confidence in the health care system’s protective mechanisms, negatively impacting their satisfaction and intention to use such systems. Additionally, physicians and pharmacists depend on cross-system data exchange and access, such as retrieving medical records from hospital information systems and transmitting prescription data to the NHIA. Without robust security measures and monitoring, health care professionals may question the controllability and accountability of patient data, compromising their satisfaction and intention to use.

From a practical implementation perspective, the government should enact regulations to establish a legislative foundation for issuing and transferring electronic prescriptions. Medical institutions should conduct regular information security audits addressing both managerial and technical aspects. Managerial tasks include providing periodic internal information security training and seminars to enhance security awareness, establishing information security management systems, and executing regular internal audits and control procedures. Technical tasks include routine vulnerability scanning and penetration testing, applying security patches, and inspecting protective mechanisms such as firewalls and intrusion detection systems.

While the statistical validation in Chapter 4 necessitated merging ‘Trust in Technology’ into the overarching ‘Digital Trust’ construct, trust in technology remains an essential dimension inherent to digital trust and a cornerstone of e-prescribing systems. Through ICTs embedded within the electronic system to reduce errors, trust in technology strengthens users’ digital trust [8], which generates positive system experiences and increases the intention to use [71,72]. When users believe that e-prescribing systems can effectively execute prescription issuance, transmission, and inquiry functions, this trust in technology enhances overall digital trust, encompassing trust in system developers, medical institutions, and the national digital health policy framework. Once digital trust is established, users are more willing to adopt the system as a daily operational tool, thereby increasing their satisfaction as its reliability meets their expectations. Moreover, digital trust further strengthens users’ intention to continue or expand their usage.

For frontline users, trust in technology not only reflects confidence in system functionality but also is a key condition for seamlessly integrating e-prescribing systems into clinical workflows. When the system operates stably, transmits prescription information in real time, and accurately displays drug information, health care professionals gradually build a sense of digital trust. For example, when physicians are confident that prescriptions completed in the consultation room are accurately transmitted to pharmacies, the burden of repeated verification or telephone confirmation can be significantly reduced. Similarly, when pharmacies receive prescriptions and can verify the accuracy of the information, they can focus on medication safety reviews and patient counseling.

Additionally, system stability and error-prevention functionalities are closely related to medical responsibility. Transmission of incorrect information may compromise patient safety and lead to medical disputes. Thus, developing trust in technology is an important measure for reducing occupational risk and psychological burden. When health care professionals view a system as trustworthy, they operate it with confidence. They are more likely to continue using it, facilitating the adoption and acceptance of the overarching digital health policy. From a practical implementation perspective, enhancing system security and stability in medical institutions and pharmacies elevates users’ trust in technology, thereby strengthening public satisfaction and intention to use. Moreover, to build user trust and support for e-prescribing systems, the government should enhance communication and education, clearly explaining the safety and value of e-prescribing systems to the public and transparently disclosing system processes and operational mechanisms [88].

From a managerial perspective, factors such as access to medicines, digital health service quality, and digital trust exert significant positive effects on user satisfaction and intention to use. Thus, the design and promotion of e-prescribing systems must focus on convenience in obtaining medications, service quality, information security and privacy, and perceived system stability. Digital trust acts as a key mediating role, linking overall system mechanisms to institutional trust and satisfaction, thereby promoting both user satisfaction and usage behavior. Meanwhile, user satisfaction plays an important mediating role between antecedent variables (access to medicines, digital health service quality, and digital trust) and outcome variables (user satisfaction and intention to use), serving as a critical driver of the continued adoption and acceptance of e-prescribing systems. Therefore, during the implementation of digital health information systems, administrators should employ integrated strategies that are both digital trust-oriented and user-oriented. For instance, medical institutions and pharmacies can optimize system connectivity and synchronization mechanisms and introduce bidirectional query functions. This enables pharmacists to confirm prescription uploads in real time while allowing patients to receive SMS or app notifications upon completion of medication preparation. Such improvements substantially enhance convenience in obtaining medications. Furthermore, digital trust and user satisfaction among physicians and pharmacists are reinforced by simplified workflows and clearer communication. On this basis, e-prescribing systems can enhance users’ access to medicines, service quality, and digital trust through system functionality, information security design, and technical integration. This fosters positive user experiences among health care professionals and facilitates sustained system use and broad policy implementation.

### 5.2. Research Limitations and Recommendations for Future Research

This study has several limitations. First, data were collected through physical questionnaires, with distribution limited by location and timing. The sample consisted of health care professionals at clinics, health centers, hospitals, and pharmacies in Hualien County, Eastern Taiwan, which may introduce sampling bias. Second, the questionnaire was designed for physicians, pharmacists, medical administrative staff, and information engineers who use e-prescribing systems. The primary limitation arises from the novelty of the e-prescribing initiative; since no prior baseline system existed, the sample size of active respondents is significantly smaller than that typically found in general information system adoption studies.

Future research should expand the cohort, for example, by gathering patient feedback to objectively examine and compare actual user experiences with patient expectations. Third, subgroup analyses by medical specialty were not conducted. Given variations across specialties in medication categories and clinical practices, future studies should conduct comparative analyses within specific specialties to enhance the breadth and depth of relevant research.

## 6. Conclusions

This study proposes an innovative technological and socio-psychological framework tailored to the unique context of electronic prescribing (e-prescribing) systems, validating the factors that drive health care professionals’ adoption intentions during the initial implementation phase. The empirical results indicate that both access to medicines and digital trust significantly influence usage intentions, with institutional trust serving as a critical determinant that shapes digital trust. Additionally, user satisfaction directly drives intention to use. Path analysis reveals key indirect relationships: institutional trust indirectly impacts both user satisfaction and usage intentions, mediated by digital trust; meanwhile, access to medicines, digital health service quality, and digital trust each exert indirect effects on intention to use through user satisfaction. Ultimately, these findings offer valuable insights for policy formulation, system design, and managerial decision-making. By enhancing the quality of experience and satisfaction for health care professionals, patients, and their families, this research supports progressive advancements in patient safety, medication management, and overall health care quality.

## Figures and Tables

**Table 1:** Subjects Recruitment and Sample Profile.

Participating Institutions	Six Medical Institutions and Eleven Community Pharmacies
Recruitment Procedure	The recruitment procedure involved engaging health care professionals in a hands-on simulation of the e-prescribing workflow, which spanned the clinical consultation, payment, and medication dispensing phases, alongside accessing relevant prescription information via a mobile application.
Participant Background (professional composition of the sample)	Physicians, pharmacists, nurses, medical administrative staff, and information engineers who had used e-prescribing systems from hospitals, public health centers, clinics, and pharmacies.
Response Rate	100%

**Table 2:** Measurement model assessment results.

Question Item	Factor Loading	SMC	Cronbach’s α	CR	AVE
Access to Medicines (ATM)			0.893	0.909	0.774
1. Patients can obtain their medications at the pharmacy through e-prescribing.	0.651	0.424			
2. Compared with paper prescriptions, I find it more convenient using e-prescribing to obtain medicines.	0.978	0.956			
3. Compared with paper prescriptions, I find that e-prescribing makes the entire medical visit process smoother.	0.971	0.943			
Digital Health Service Quality (DHSQ)			0.916	0.916	0.786
1. After consultation, the physician clearly answers the patient’s questions on the electronic prescription.	0.819	0.671			
2. When I am obtaining medications at the pharmacy, the pharmacist clearly answers my questions regarding the electronic prescription.	0.897	0.805			
3. Overall, the service quality of e-prescribing is good.	0.939	0.882			
Institutional Trust (IT)			0.920	0.930	0.869
1. I believe e-prescribing ensures the security of personal information.	0.922	0.851			
2. I believe e-prescribing protects personal privacy.	0.942	0.887			
Trust in Technology (TIT)			0.943	0.946	0.853
1. I believe that e-prescribing allows pharmacies to verify medications quickly and reduce errors.	0.904	0.817			
2. I feel assured when using e-prescribing.	0.946	0.895			
3. E-prescribing makes it easier for patients to obtain medicines.	0.920	0.846			
Digital Trust (DT)			0.979	0.979	0.921
1. I believe the quality of e-prescribing is high.	0.932	0.869			
2. E-prescribing is reliable.	0.968	0.937			
3. E-prescribing meets my health care needs.	0.956	0.914			
4. Overall, I trust e-prescribing.	0.982	0.964			
User Satisfaction (US)			0.980	0.979	0.939
1. I find the e-prescribing QR code very convenient.	0.940	0.884			
2. Compared with paper prescriptions in the past, I am satisfied with using e-prescribing to obtain medicines.	0.979	0.958			
3. Overall, I am satisfied with using e-prescribing.	0.987	0.974			
Intention to Use (IU)			0.973	0.973	0.922
1. I am willing to use e-prescribing.	0.921	0.849			
2. I would like to recommend e-prescribing to others.	0.971	0.943			
3. My evaluation of e-prescribing is positive.	0.988	0.976			

**Table 3:** Results of discriminant validity analysis.

Construct	ATM	DHSQ	IT	DT	US	IU
1. ATM	0.88					
2. DHSQ	0.82 ***	0.89				
3. IT	0.72 ***	0.79 ***	0.93			
4. DT	0.83 ***	0.87 ***	0.87 ***	0.94		
5. US	0.87 ***	0.89 ***	0.79 ***	0.90 ***	0.97	
6. IU	0.87 ***	0.84 ***	0.82 ***	0.92 ***	0.92 ***	0.96

Note: ATM: Access to Medicines; DHSQ: Digital Health Service Quality; DT: Digital Trust; US: User Satisfaction; IU: Intention to Use; IT: Institutional Trust; *** *p* < 0.001; the diagonal is the square root of the AVE value of each construct, while the non-diagonal values are correlation coefficients between two constructs.

**Table 4:** Summary of Standardized Path Coefficients and Hypothesis Testing Results of the Research Model.

Hypothesis	Path	Path Coefficient	*p*-Value	Hypothesis Supported
H1a	ATM → US	0.381 ***	0.000	Supported
H1b	ATM → IU	0.286 ***	0.000	Supported
H2a	DHSQ → US	0.411 ***	0.000	Supported
H2b	DHSQ → IU	−0.168	0.168	Not Supported
H3	IT → DT	0.981 ***	0.000	Supported
		-	-	NA (TIT and DT were merged into “DT”)
H5a	DT → US	0.206 *	0.018	Supported
H5b	DT → IU	0.449 ***	0.000	Supported
H6	US → IU	0.432 ***	0.000	Supported

Note: ATM: Access to Medicines; DHSQ: Digital Health Service Quality; DT: Digital Trust; US: User Satisfaction; IU: Intention to Use; IT: Institutional Trust; TIT: Trust in Technology; NA: Not Available; * *p* < 0.05; *** *p* < 0.001.

**Table 5:** Mediation Analysis Results.

IV, M, DV	IV → M (a)	M → DV (b)	IV → M → DV (a × b)	Bootstrapping 95% CI (Bias Corrected)
IT → DT → US	0.914 ***	0.885 ***	0.809 ***	0.461~1.097
IT → DT → IU	0.914 ***	0.790 ***	0.722 ***	0.342~1.072
ATM → US → IU	0.879 ***	0.655 ***	0.576 ***	0.396~0.770
DHSQ → US → IU	0.891 ***	0.896 ***	0.798 ***	0.655~0.956
DT → US → IU	0.902 ***	0.517 ***	0.466 ***	0.257~0.673

Note: ATM: Access to Medicines; DHSQ: Digital Health Service Quality; DT: Digital Trust; US: User Satisfaction; IU: Intention to Use; IT: Institutional Trust; IV: independent variable; M: mediator; DV: dependent variable; CI: confidence interval; *** *p* < 0.001. The coefficients in columns 2 through 4 are all standardized coefficients.

## Data Availability

The data presented in this study are available on request from the corresponding author. The data are not publicly available due to privacy and ethic restriction.
